# Bone Marrow-Derived Mesenchymal Stem Cells Ameliorate Hepatic Ischemia Reperfusion Injury in a Rat Model

**DOI:** 10.1371/journal.pone.0019195

**Published:** 2011-04-29

**Authors:** Hiroyuki Kanazawa, Yasuhiro Fujimoto, Takumi Teratani, Junji Iwasaki, Naoya Kasahara, Kouji Negishi, Tatsuaki Tsuruyama, Shinji Uemoto, Eiji Kobayashi

**Affiliations:** 1 Division of Development of Advanced Treatment, Center for Development of Advanced Medical Technology, Jichi Medical University, Yakushiji City, Tochigi, Japan; 2 Department of Diagnostic Pathology, Kyoto University Hospital, Kyoto City, Kyoto, Japan; 3 Department of Hepato-pancreato-biliary Surgery and Transplantation, Graduate School of Medicine, Kyoto University, Kyoto City, Kyoto, Japan; Istituto Dermopatico dell'Immacolata, Italy

## Abstract

**Background:**

Ischemia-reperfusion (I/R) injury associated with living donor liver transplantation impairs liver graft regeneration. Mesenchymal stem cells (MSCs) are potential cell therapeutic targets for liver disease. In this study, we demonstrate the impact of MSCs against hepatic I/R injury and hepatectomy.

**Methodology/Principal Findings:**

We used a new rat model in which major hepatectomy with I/R injury was performed. Male Lewis rats were separated into two groups: an MSC group given MSCs after reperfusion as treatment, and a Control group given phosphate-buffered saline after reperfusion as placebo. The results of liver function tests, pathologic changes in the liver, and the remnant liver regeneration rate were assessed. The fate of transplanted MSCs in the luciferase-expressing rats was examined by in vivo luminescent imaging. The MSC group showed peak luciferase activity of transplanted MSCs in the remnant liver 24 h after reperfusion, after which luciferase activity gradually declined. The elevation of serum alanine transaminase levels was significantly reduced by MSC injection. Histopathological findings showed that vacuolar change was lower in the MSC group compared to the Control group. In addition, a significantly lower percentage of TUNEL-positive cells was observed in the MSC group compared with the controls. Remnant liver regeneration rate was accelerated in the MSC group.

**Conclusions/Significance:**

These data suggest that MSC transplantation provides trophic support to the I/R-injured liver by inhibiting hepatocellular apoptosis and by stimulating regeneration.

## Introduction

Liver transplantation is one of the most efficient treatments available for various end-stage hepatic diseases. However, one of the major limitations of liver transplantation is the scarcity of donor organs. To overcome this limitation, split liver transplantation or living donor liver transplantation are performed and show the most promising outcomes. However, when these treatments are performed in adult recipients with adult living donors, size mismatch between graft and recipient becomes a critical problem. The first priority is donor safety, and so smaller sized grafts such as the left-lobe of the liver seem to be the optimal choice [1].

Liver transplantation does inevitably lead to hepatic ischemia-reperfusion (I/R) injury. Primary graft non-function or dysfunction, which occurs as a result of combined I/R injury and secondary tissue regeneration impairment, remains a serious complication in the clinical practice, and this is especially the case for transplantation of livers that are small-for-size [2], [3]. Therefore, many studies have tried to elucidate the mechanisms of I/R injury by means of appropriate in vivo models. Effective treatment strategies aimed at reducing hepatic I/R injury and accelerating liver regeneration could offer major benefits in liver transplantations involving size mismatch of graft and recipient.

Recent reports have demonstrated the capacity of mesenchymal stem cells (MSCs) to specifically be involved in the repair of organ tissue. These results indicate that MSCs are an attractive cell source for regenerative medicine. As for the liver, most animal studies have been carried out in drug-induced rodent models [4], [5], [6]. However, the role of MSCs in hepatic I/R injury remains to be established.

Here, we show that transplanted MSCs are able to ameliorate hepatic I/R injury and significantly improve liver regeneration.

## Results

### Characterization of BM-MSCs

**Figure 1:**
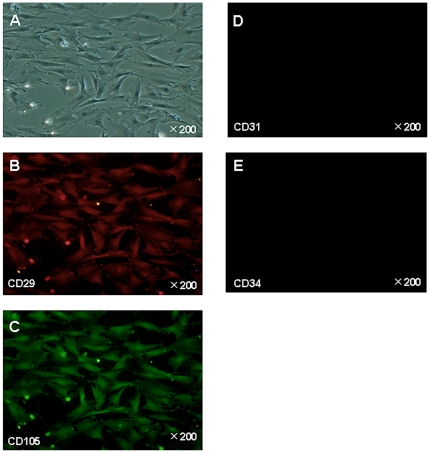
Characterization of BM-MSCs by expression of CD29^+^, CD31^−^, CD34^−^, and CD105^+^. **A**. Bright-field image. **B–C**. CD29 and CD105 surface antigens are positive. **D**. CD31 surfice antigen is negative, **E**. CD34 surfice antigen negative.

**Figure 2:**
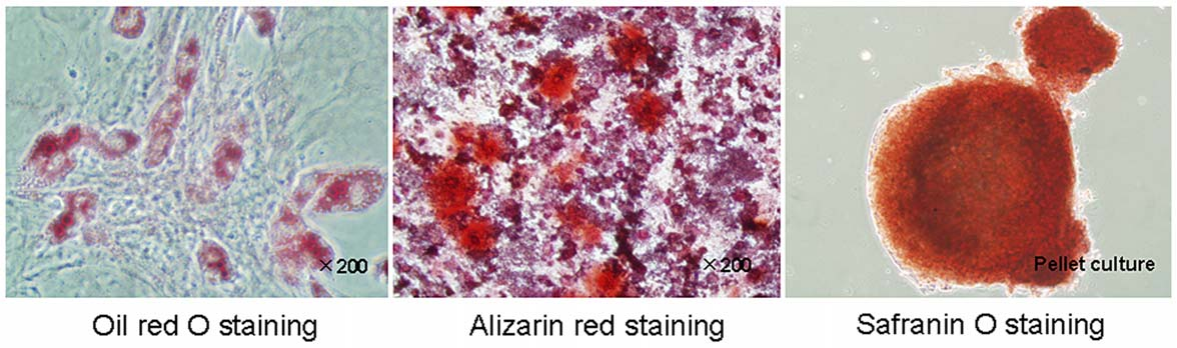
Characterization of BM-MSCs. BM-MSCs at passage 5 are induced to differentiate into adipocytes, osteoblasts, and chondrocytes-like cells. Cells analyzed by cytochemical staining with Oil Red-O, Alizarin red, or Safranin-O, respectively.

Bone marrow-derived mesenchymal stem cells (BM-MSCs) were spindle shaped and plastic-adherent cells in standard culture conditions (Figure 1A). BM-MSCs were characterized by immunofluorescence. We identified the expression of CD29^+^, CD105^+^, CD31^−^, and CD34^−^ on BM-MSCs by immunostaining (Figure 1B, 1C, 1D, 1E). Previous data showed that rat MSCs were positive for CD29 and CD105, but were negative for CD31 and CD34 [7], [8]. Differentiation ability of BM-MSCs into classical mesenchymal lineage cells including adipocytes, osteoblasts, or chondrocytes was verified by using previously reported methods (Figure 2). The results indicated that the cells were undifferentiated and had stem cell characteristics.

### Effect of BM-MSCs on increased levels of serum ALT induced by I/R in rats

Serum AST and ALT levels were measured to determine damage to hepatocytes 24 h after reperfusion. Serum ALT levels were significantly decreased in the MSC group compared with the Control group (MSC group: 1136±325 IU/L, Control group: 2198±854 IU/L, *P*<0.03). Serum AST levels were also decreased (MSC group: 1043±223 IU/L, Control group: 1377±428 IU/L, but not significantly). However, liver sections of the MSC group were not morphologically distinguishable (H&E) from sections of the Control group (data not shown).

### Decreased luciferase activity in MSC rats

**Figure 3:**
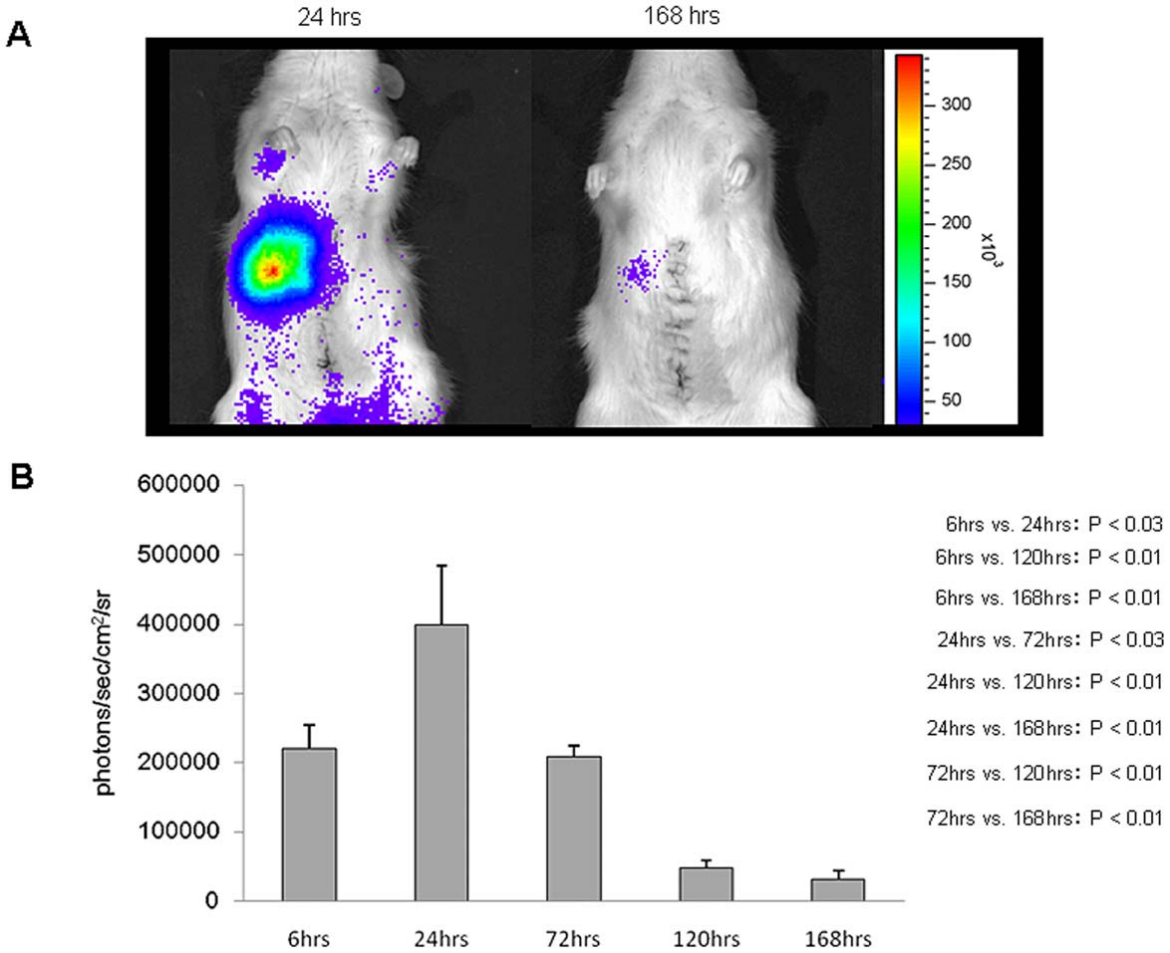
Decrease of luciferase activity. The expression level of luciferase was postoperatively observed using a noninvasive living image acquisition IVIS system. Accumulation of MSCs in the remnant liver. **A**. Most of the MSCs became trapped in the remnant liver. Thereafter, the luciferase activity diminished with time. **B**. The largest level of luciferase was 187272±119507 photons/sec/cm^2^/sr (sr = units of solid angle or steradian). (24 h vs. 168 h: *P*<0.03; 24 h vs. 120 h: P<0.05; 72 h vs. 168 h: P<0.05).

Twenty-four hours after reperfusion, most of the MSCs were detected in the remnant liver by In Vivo Imaging System (IVIS), and thereafter, luciferase activity diminished with time (Figure 3). When luciferase was measured 168 h after reperfusion, the amount of light emitted was only slightly detectable.

### BM-MSC transplantation inhibits apoptosis after I/R injury

**Figure 4:**
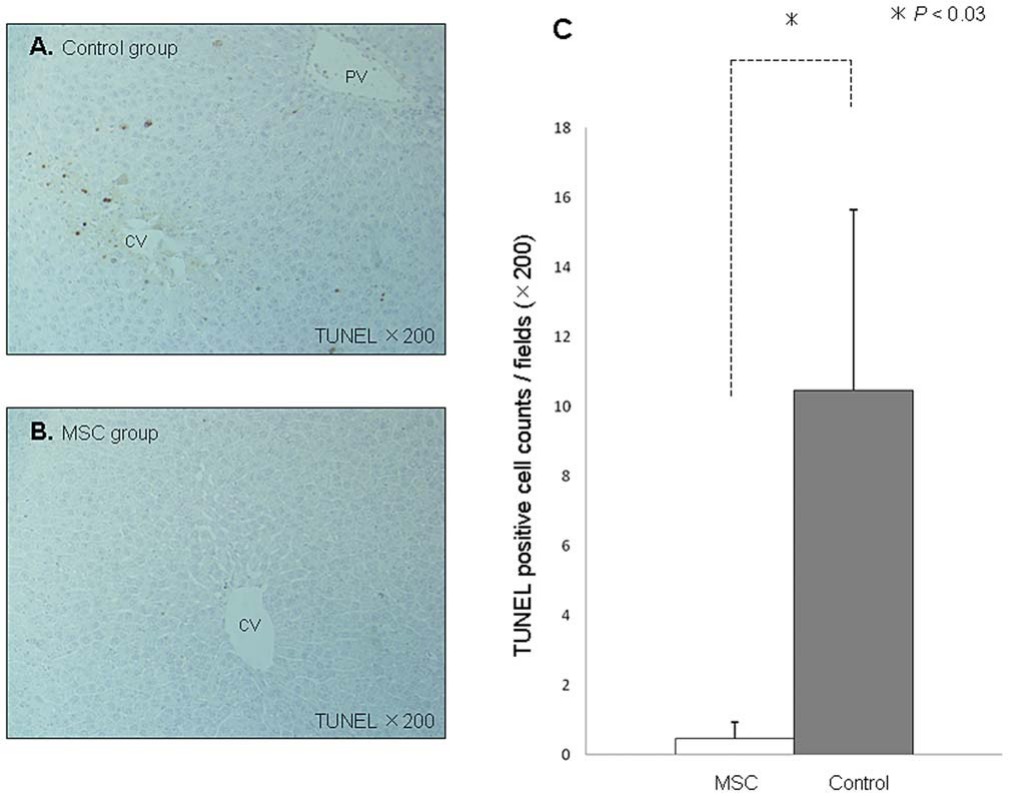
Minimal TUNEL-positive hepatocytes by BM-MSC transplantation. TUNEL staining of I/R-injured liver sections from the MSC group (**A**. upper panel) and the Control group (**B**. lower panel). **C**. Quantification of TUNEL-positive hepatocyte nuclei was assessed by calculating the mean of the number of TUNEL-positive hepatocytes in 10 random high-power fields per animal. Abbreviations: CV, central vein; PV, portal vein.

TUNEL-positive hepatocytes in the Control group were mainly localized in the centrilobular region (Figure 4A). The extent of hepatocyte apoptosis was evaluated by TUNEL staining 6 h after reperfusion. TUNEL staining yielded a mean of 10.5±0.7 positively stained cells/high power field in the Control group, and 0.5±0.5 in the MSC group (*P*<0.03) (Figure 4C).

### X-gal immunostaining reveals localization of BM-MSCs in the liver

**Figure 5:**
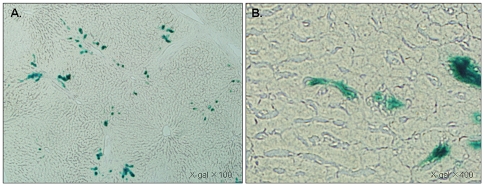
LacZ-positive BM-MSCs distributed around the periportal area. Photomicrograph of liver sections stained with X-gal from a rat 6 h after reflow. **A**. LacZ-positive MSCs (blue) detected around the periportal area. **B**. MSCs detected in the sinusoid (right panel).

Almost all of the LacZ-positive BM-MSCs were distributed around the portal triad and interlobular connective tissue 6 h after reperfusion (Figure 5A). There were very few LacZ-positive BM-MSCs in the centrilobular region. When the periportal area was examined more closely, BM-MSCs were also detected in the sinusoids (Figure 5B).

### BM-MSC administration promotes the rate of liver regeneration

**Figure 6:**
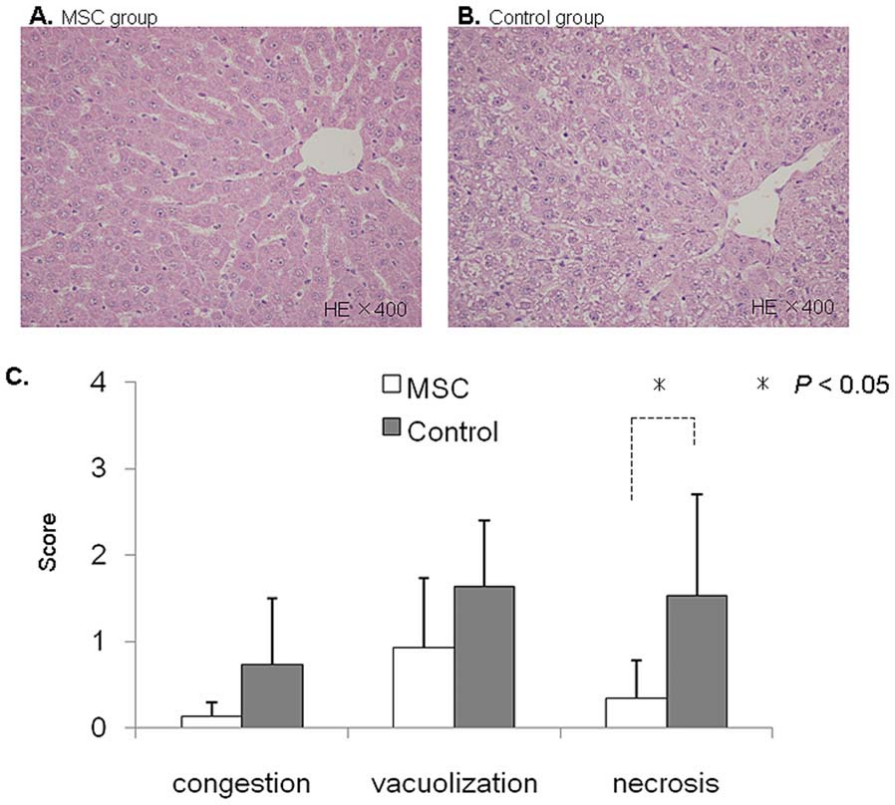
Histopathological changes and Suzuki Score. Hematoxylin eosin staining of I/R-injured liver sections from the MSC group (**A**. left panel) and the Control group (**B**. right panel). **C**. The lower levels of congestion, vacuolization, and necrosis were seen in the MSC group. Suzuki scores = (MSC group vs. Control group: 1.4±1.3 vs. 3.9±1.7, *p*<0.03).

After the operative procedure, all of the rats survived until sacrifice. Seven-two hours after reperfusion, H&E staining revealed significant morphological changes in Suzuki scores (1.4±1.3 vs. 3.9±1.7, *p*<0.03) and necrosis scores (0.3±0.5 vs. 1.5±1.2, *p*<0.05) in the MSC group compared with the Control group (Figure 6). As shown Figure 6A–B, the pathological findings revealed less vacuolar degeneration in the MSC group compared with the Control group. However, these pathological changes had almost completely improved 168 h after reperfusion, as the H&E-stained liver tissues were no longer morphologically distinguishable between the two groups (data not shown).

**Figure 7:**
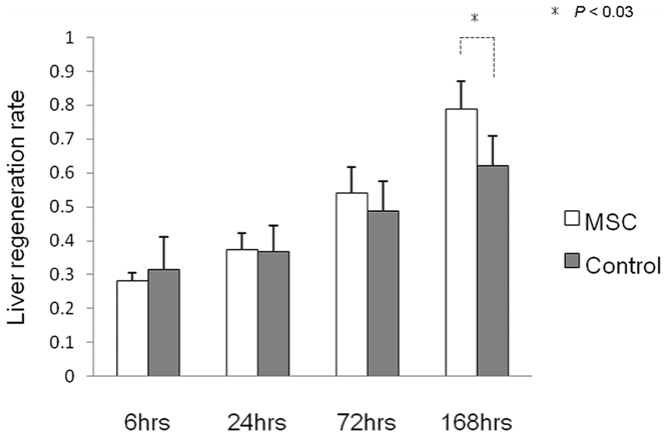
Remnant liver volume changes. The liver regeneration rate is expressed as remnant liver weight/estimated whole liver weight.

To examine the recovery of the remnant liver, we assessed the liver regeneration rate. As shown in Figure 7, the liver regeneration rate in the MSC group was significantly increased 168 h after reperfusion compared with the Control group (*P*<0.03).

## Discussion

BM-MSCs have the capacity for self-renewal and multipotency, and can differentiate into bone, fat and cartilage cells [9], [10]. Cell-based therapy has recently been considered a prominent tool in regenerative medicine. However, the use of embryonic stem cells or induced pluripotent stem cells is ethically controversial and can also result in teratoma formations that hinder the applications of stem cell therapy. One way to circumvent these issues is to use MSCs, as they are an attractive alternative for regenerative medicine.

In recent years, MSC transplantation has been broadly used in animal models of cerebral infarction, myocardial infarction, and renal I/R injury to regenerate damaged tissues. Some reports have published the effectiveness of MSC transplantation against drug-induced liver injury [4], [5]. However, the effectiveness of MSC transplantation against hepatic I/R injury remains unknown. Hepatic I/R injury results in pathological changes such as congestion, vacuolization, and apoptosis in liver tissue, which can lead to hepatic failure.

Fatal complications in patients receiving major hepatectomy by the Pringle maneuver or by liver transplantation with small-for-size grafts can be reduced by minimizing hepatic I/R injury and avoiding the progress of harmful events following I/R injury.

### New animal model

In the present study, our rat model provided the ideal animal model to research I/R injury after major hepatectomy by the Pringle maneuver or liver transplantation with small-for-size grafts. Intestinal congestion was avoided during the application of hepatic ischemia by bypassing portal flow through nonischemic lobes (right and caudate lobes). Moreover, the left lateral and the left portion of the medial lobes as well as the nonischemic lobes were excised at the onset of reflow to mimic the clinical condition of liver transplantation with small-for-size grafts or major hepatectomy by the Pringle maneuver in order to research postischemic liver functions.

### The optimal route of transplantation and the optimal number of MSCs

We selected the portal vein as the route for MSC transplantation. Systemically transplanted MSC photons were monitored using IVIS™, and were mostly trapped in the microvasculature of the lung because of their size and adhesion potential. Moreover, the MSC-derived photons from the lung faded out within 1 day, and no photons in the I/R-injured liver were detected (data not shown). It is unlikely that recirculating MSCs after pulmonary trapping migrated to the I/R-injured liver. Thus, the systemic route is not always considered the best route for MSC transplantation despite the migration potential of MSCs. A better method for MSC delivery to the liver might be through the portal vein.

The optimal number of MSCs is a critical factor, yet little is known about the optimal cell dose. Most reports have used doses of 2 to 10 million MSCs per kilogram in small animal experiments [11]. Some reports have shown that the greater the dose of MSCs transplanted, the greater the therapeutic effect that is achieved. However, the maximum dose in rats and mice is determined by the number of cells that do not undergo fatal embolism by transplantation (usually not more than 10 million cells overall).

### Therapeutic effect of MSCs against liver injury

In a model of drug-induced chronic liver disease, a previous report has shown that transplanted MSCs can be involved in anti-fibrotic effects. The authors also showed that transplanted MSCs scattered mostly in the hepatic connective tissue and survived in the liver 4 weeks after transplantation, but did not differentiate into hepatocytes expressing albumin or alpha-fetoprotein [5]. This report suggested that a variety of bioactive cytokines secreted by the transplanted MSCs might be involved in restoring liver function and promoting regeneration. In addition, another report has shown that MSC-conditioned medium has the potential to dramatically reduce cell death [12], [13].

On the other hand, certain studies have described the transdifferentiation of MSCs into cells with a hepatocyte-like phenotype [14], [15], [16], [17]. Models of chronic liver disease have a relatively long time for the onset of cell therapy effects to be established, and the period of observation is also relatively longer after the MSCs are transplanted. It is still controversial whether transplanted MSCs protect and regenerate the liver by cell fusion or by transdifferentiation, or by neither. Thus, further studies on the fate of MSCs after transplantation are necessary.

In models of acute liver disease due to I/R injury, free radicals generated during the acute phase of I/R injury initiate the inflammatory cascade, giving rise to the second attack, which is characterized by infiltration of activated neutrophils in the liver promptly after reperfusion. Activation of Kupffer cells and T lymphocytes promotes neutrophil recruitment, assisted by increased endothelial expression of adhesion molecules. Therefore, transplanted MSCs need to work efficiently shortly after reperfusion. In the present study, MSC treatment ameliorated the increase in serum transaminase levels, which serves as the most sensitive marker for clinical and experimental hepatic I/R injury evaluation. These findings indicate that MSCs were viable and able to function shortly after transplantation. There is limited time for MSCs to transdifferentiate into hepatocytes or hepatocyte-like cells during the acute phase. Therefore, it is unlikely that transdifferentiation is involved in tissue protection and repair.

Histopathological findings showed that hepatocyte apoptosis induced by I/R injury mainly existed in the centrilobular region in the Control group, whereas LacZ-positive MSCs were detected in the periportal area in the MSC group. Transplanted MSCs did not replace damaged hepatocytes, but settled down in the periportal area. This evidence supports the notion that paracrine actions exerted by MSCs through the release of soluble factors might be important for tissue protection and repair. Moreover, the luciferase activity of MSCs gradually decreased and at 168 h after reperfusion, we could not find any LacZ-positive cells in the liver sections (data not shown). Once the liver fully recovers, it is possible that host hepatocytes identify the transplanted MSCs as non-self cells and eliminate them from the liver. Seventy-two hours after reperfusion, a degree of liver injury was improved in the MSC group compared to the Control group. It seems reasonable that MSC transplantation attenuating I/R injury would result in the histopathological differences observed between the MSC group and the Control group. Also, the sequence of events might affect liver regeneration and cause a significant difference in remnant liver regeneration 168 h after reperfusion.

In conclusion, these findings suggest that MSCs might have the potential to protect the liver against I/R injury-induced hepatocyte apoptosis, and to enhance liver regeneration.

## Materials and Methods

### Animal

All experiments were conducted under the approval of the Jichi Medical University Guide for Laboratory Animals (Approval number: # 1080).

Male wild Lewis rats were purchased from Charles River (Breeding Laboratories, Kanagawa, Japan). Rats used in the experiments had a body weight of between 230 and 310 g.

The animals were housed in a temperature- and humidity-controlled environment with a 12 h light/12 h dark cycle with free access to food (standard laboratory chow) and water *ad libitum*. After fasting overnight, all animals were anesthetized with ether inhalation.

### Establishment of double transgenic rats

Double transgenic (Tg) rats expressing luciferase and LacZ were created by crossbreeding ROSA/luciferase Tg Lewis rats [18] with ROSA/LacZ Lewis rats [19]. IVIS was used to detect luciferase expression, and X-gal staining was used to detect LacZ expression (detailed below). The F1 hybrids between ROSA/luciferase Tg and ROSA/LacZ Lewis rats were imaged after intravenous injection of D-luciferin (30 mg/kg/body weight) (potassium salt; Bio-synth, Postfach, Switzerland), followed by X-gal staining. In the same manner, luciferase and LacZ expression levels were examined in various tissues of the rats. Approximately one-fourth of the F1 hybrids expressed luciferase and LacZ in the whole body. We used these “dual colored” F1 hybrids expressing both luciferase and LacZ (luc/LacZ) as our MSC donors.

### Bone marrow-derived MSC preparation and culture

Bone marrow cells were isolated from female double (luc/LacZ) Tg rat femurs by flushing the femurs with αMEM (invitorgen, Tokyo) supplemented with 10% fetal bovine saline (invitrogen, Tokyo) and antibiotic-antimycotic (invitrogen, Tokyo), using a 19-gauge needle. Isolated bone marrow cells were seeded onto 10-ml tissue culture dishes (Thermo Scientific, Tokyo), and cultured with αMEM supplemented with 10% FBS. When the cells were 70%–80% confluent, they were harvested with 0.05% trypsin-EDTA (invitrogen, Tokyo), replated at 2×10^4^ cells/cm^2^, and cultured for 5 days. MSCs between the fifth and eighth passage were used for the experiments.

### Operative procedure

**Figure 8:**
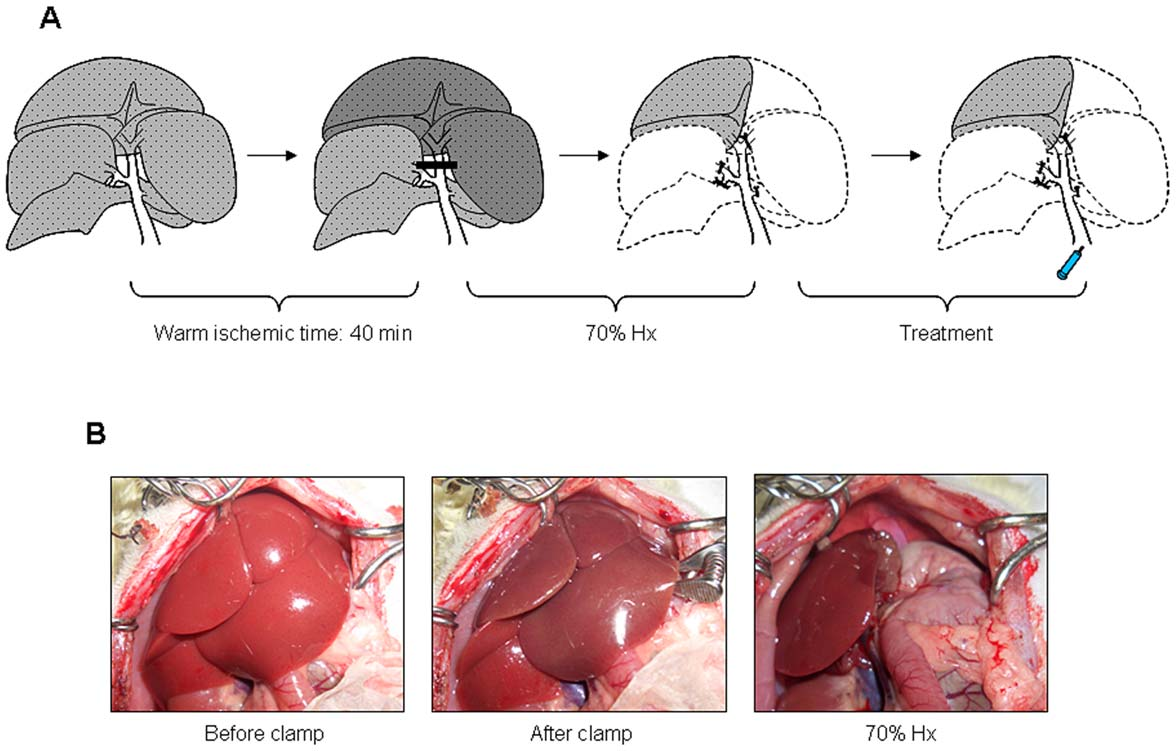
A new rat model of major hepatectomy with I/R injury.

A temporary warm ischemia of the liver was induced as shown in Figure 8. All surgical procedures were performed under light ether anesthesia. The abdomen was opened through a midline incision and ligaments surrounding each lobe were then dissected away. Clamping the portal vein, the hepatic artery, and the bile duct supplying the median and the left lateral lobes of the liver with a microvessel clip induced 70% partial liver ischemia. This technique enabled us to avoid intestinal congestion, which may lead to fatal hemodynamic instability [20], [21]. After 40 min of partial hepatic ischemia, the vessel clip was released to initiate hepatic reperfusion. Immediately after onset of reperfusion, the nonischemic lobes (the superior right lateral, the inferior right lateral, the anterior caudate and the posterior caudate lobes) were excised. Furthermore, these procedures were followed by left lateral and left portion of the medial lobectomy, leaving only the ischemic right portion of medial lobe behind. Subsequently, phosphate-buffered saline (PBS) or 1×10^6^ MSCs in a volume of 200 µl were transfused into the portal vein with a 30-gauge needle for over 1 min. The abdomen was closed with 3-0 silk sutures, and the animals were allowed to awaken and then given free access to food and water.

Animals were sacrificed after the reperfusion of 6, 24, 72, and 168 h (n = 6 per each point). Blood samples were taken from the inferior vena cava for liver function tests, and the liver was harvested and weighed. Liver tissue samples were collected and properly preserved for subsequent procedures.

### Immunofluorescence

Cells were fixed in 4% formaldehyde for 10 min, followed by incubation with Protein Block for 30 min. Bone marrow-derived MSCs were analyzed by immunohistochemistry using monoclonal anti CD29 (VMRD, Inc., USA), CD31 (santa cruz biotechnolog, Inc., USA), CD34 (R&D Systems, USA), and CD105 (santa cruz biotechnology, USA) antibodies overnight at 4°C [22], [23]. The fluorescein (green, 1∶3000)-conjugated secondary antibody (COSMO BIO, Inc., Tokyo) against CD31, CD34, and CD105 was applied for 30 min. Rhodamine (red, 1∶3000)-conjugated secondary antibody (Rockland immunochemicals, Inc., Tokyo) against CD29 was also used.

### Evaluation of the mesenchymal lineage differentiation of MSCs

The differentiation potential of MSCs (passage 5) into adipocytes, osteocytes, or chondrocytes was evaluated using differentiation-induction media purchased from Lonza Walkersville, Inc. (http://www.lonza.com) according the manufacturer's protocols.

### Detection and quantification of transgene expression using a non-invasive in vivo imaging system

In Vivo Imaging System IVIS™ (Xenogen, Allameda, CA) was used for the analysis of luciferase gene expression activity. In this system, a noninvasive charged-couple device camera was used to detect bioluminescene emitted from *D*-luciferin, which reacts with firefly luciferase in living animals. While under isoflurane anesthesia, the rats received *D*-luciferin through the penile vein [30 mg/kg/body weight, dissolved and diluted to 15 mg/ml in PBS] [24]. Immediately after the infusion, the light emitted by luciferase was measured, with a 1-min integration time. The signal intensity was quantified as photon flux in units of photons/sec/cm^2^/steradian in the region of interest [25]. The results show the mean values of 3 rats in each of 6, 24, 72, 120, and 168 h after reperfusion.

### Assessment of liver functions

Blood samples were obtained from each rat and centrifuged for 10 min at 3,000 rpm, and serum was collected. Concentration of markers of liver injury such as GOT and GPT was analyzed using a FUJIFILM DRI-CHEM 3500 machine (FujiFilm, Tokyo, Japan; http://www.fujifilm.co.jp) and FUJI DRY CHEM SLIDES (FujiFilm), respectively, for GOT/asparatate aminotransferase (AST)-PIII and GPT/alanine transaminase (ALT)-PIII.

### Histological analysis

After euthanasia, livers were fixed with 10% buffered formalin for paraffin embedding, or in OCT compound for frozen sections with no fixation. Five-micron paraffine-embedded sections were stained with hematoxyline and eosin (H&E) for conventional morphological evaluation. Suzuki classification [26], which consisted of 3 parameters of hepatic ischemia reperfusion injury: sinusoidal congestion, vacuolization of hepatocyte cytoplasm, and parenchymal necrosis. Each parameter was graded numerically as follows: congestion: 0 = none, 1 = minimal, 2 = mild, 3 = moderate, and 4 = severe. The same criteria were utilized in the graduation of the vacuolization, and for necrosis, the numerical graduation was as follows: 0 = nonnecrotic cells, 1 = single cell necrosis, 2 = <30% necrosis, 3 = <60% necrosis, and 4 = >60% necrosis.

### Immunohistochemical detection of LacZ-positive cells and apoptosis

To detect injected LacZ-positive MSCs in the liver, the experimental rats were sacrificed 6 h after reperfusion. Thin frozen sections (10 µm) of the liver were fixed in 0.2% glutaraldehyde for 10 min at room temperature and incubated in a solution (X-gal) containing 1 mg/ml 5-bromo-4-chloro-3-indolyl β-D-galactopyranoside (X-gal; Sigma-Aldrich, USA), 5 mM K_3_Fe(CN)_6_, 5 mM K_4_Fe(CN)_6_, and 2 mM MgCl_2_ in PBS at 37°C for 20 h.

Paraffin sections of livers sacrificed 6 h after reflow were prepared. Apoptosis was determined by in situ detection of DNA fragmentation using terminal deoxynucleotidyl transferase-mediated 2′-deoxyuridine 5′-triphosphate nick-end labeling (TUNEL) assay. Quantification of TUNEL-positive hepatocyte nuclei was assessed by calculating the mean of the number of TUNEL-positive hepatocytes in 10 random 200× fields per animal.

### Liver regeneration rate

To estimate the recovery rate of rat liver weight after partial hepatectomy, the resected liver was weighed at the time of partial hepatectomy, and when the rats were sacrificed the remnant liver was excised and weighed. The liver regeneration rate was expressed as remnant liver weight/estimated whole liver weight. The original whole liver weight was extrapolated by calculating the resected liver weight/0.663, based on the results of a pilot study that the resected liver weight in our model was equal to 66.3% of the original whole liver weight (66.3±5.5%, n = 6).

### Statistical analysis

The results are given as the mean ± SD. Statistical analysis was conducted using the student t-test for continuous data and the Mann-Whitney test for discontinuous data. A *P* value of <0.05 was considered to be statistically significant.
